# Supplementary material for: Topological antichiral surface states in a magnetic Weyl photonic crystal
Source: Nat Commun. 2023 Apr 8;14:1991. doi: 10.1038/s41467-023-37710-7 (PMC10082803; doi:10.1038/s41467-023-37710-7)
Supplement: Supplementary file 1 — Supplementary Information [file 41467_2023_37710_MOESM1_ESM.pdf]

Supplementary Information for "Topological antichiral surface states in a magnetic Weyl photonic crystal"

Xiang Xi[1†], Bei Yan[1†], Linyun Yang[1†], Yan Meng[1], Zhen-Xiao Zhu[1], Jing-Ming Chen[1], Ziyao Wang[1], Peiheng Zhou[2], Perry Ping Shum[1], Yihao Yang[3], Hongsheng Chen[3], Subhaskar Mandal[4], Gui-Geng Liu[4]*, Baile Zhang[4,5]*, Zhen Gao[1]*

[1]Department of Electronic and Electrical Engineering, Southern University of Science and Technology; Shenzhen 518055, China.

[2]National Engineering Research Center of Electromagnetic Radiation Control Materials, Key Laboratory of Multi-spectral Absorbing Materials and Structures of Ministry of Education, University of Electronic Science and Technology of China; Chengdu 611731, China.

[3]Interdisciplinary Center for Quantum Information, State Key Laboratory of Modern Optical Instrumentation, ZJU-Hangzhou Global Science and Technology Innovation Center, College of Information Science and Electronic Engineering, ZJU-UIUC Institute, Zhejiang University; Hangzhou 310027, China.

[4]Division of Physics and Applied Physics, School of Physical and Mathematical Sciences, Nanyang Technological University; Singapore 637371, Singapore.

[5]Centre for Disruptive Photonic Technologies, The Photonics Institute, Nanyang Technological University; Singapore 639798, Singapore.

†These authors contributed equally to this work.

*Corresponding author. Email: guigeng001@e.ntu.edu.sg (G.L.); blzhang@ntu.edu.sg (B.Z.); gaoz@sustech.edu.cn (Z.G.)

This PDF file includes:

Supplementary Text

Figs. S1 to S12

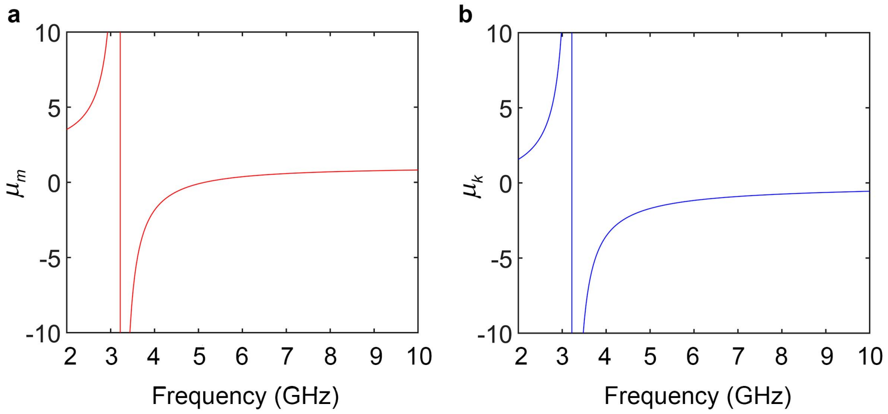

Fig. S1 | Permeability tensor of the gyromagnetic material. a, b Frequency-dependent elements μ_m and μ_k of the permeability tensor of the gyromagnetic material for $\mu_0 H_0 = 0.115$ T.

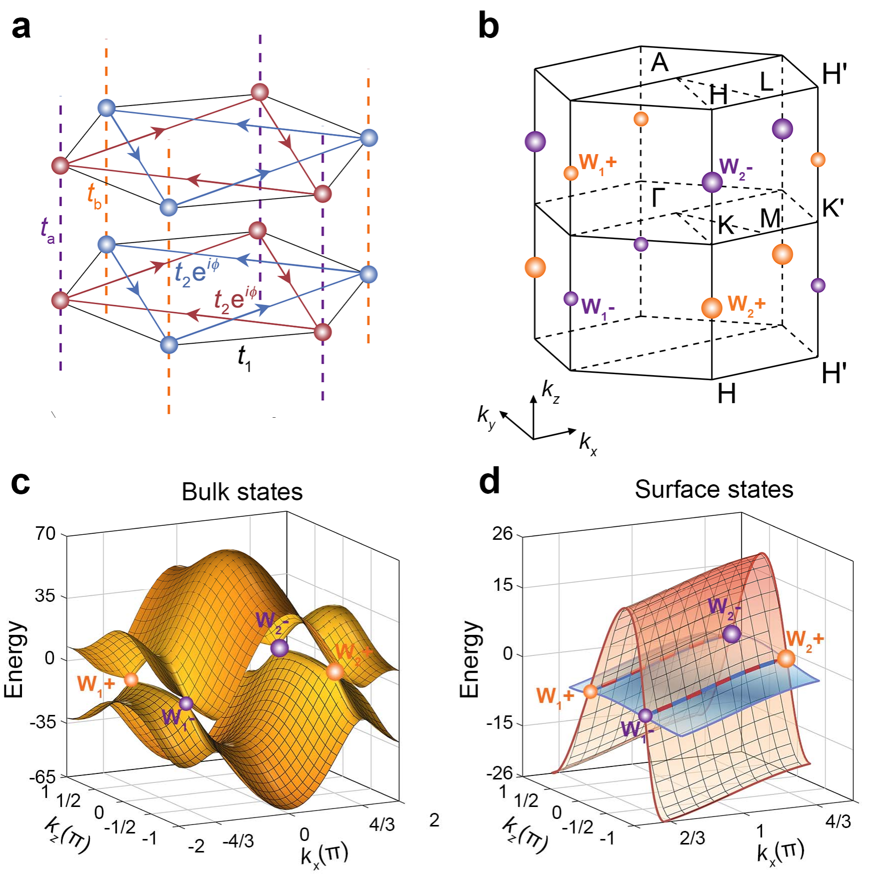

Fig. S2 | 3D modified Haldane model. a Schematic of the 3D modified Haldane model with nearest-neighbor (NN) coupling ($t_1 = 18$), next-nearest-neighbor (NNN) coupling ($t_2 = 1$ and $\phi = -\pi/3$), and interlayer couplings ($t_a = 1$ and $t_b = 10$), respectively. The red and blue spheres represent the A and B sublattice sites. **b** Three-dimensional BZ and the distribution of WPs. **c** Calculated bulk band structure of the 3D modified Haldane model, where orange and purple spheres represent two pairs of energy-shifted WPs with opposite topological charges of +1 and −1, respectively. **d** Calculated 2D surface dispersions of A-type (blue sheet) and B-type (red sheet) surfaces.

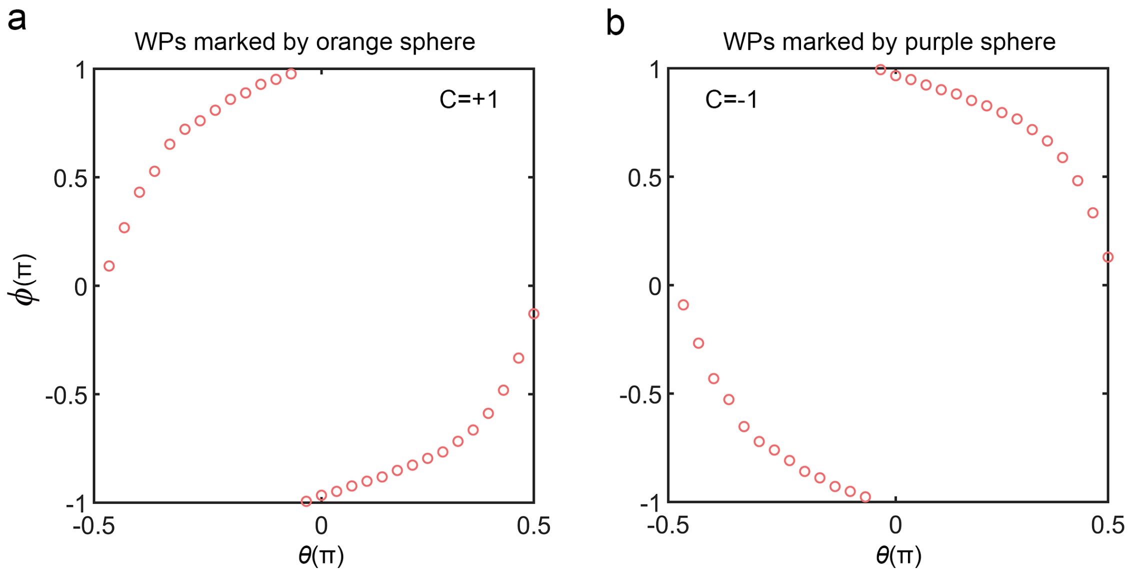

Fig. S3 | Evolution of the Berry phase on the spheres enclosing WPs with charge +1 and charge -1. Berry phase for **a** WPs with charge +1 WP at momenta (k_x, k_y, k_z) = (4π/3*a*, 0, -0.38π/*h*) and **b** WPs with charge -1 at momenta (k_x, k_y, k_z) = (4π/3*a*, 0, +0.38π/*h*), respectively.

Iso-energy contours and tilted surface dispersions of antichiral surface states in 3D modified Haldane model. Fig. S4a-c and Fig. S4d-f show the iso-energy contours of the antichiral surface states calculated by the 3D modified Haldane model on the A-type and B-type surfaces, respectively. For the A-type surface, as shown in Fig. S4a-c, its surface state iso-energy contours evolve from a single open Fermi arc connecting two projected WPs (Fig. S4a) to a single surface Fermi loop winding around the surface Brillouin zone (Fig. S4b), and finally return to a single open Fermi arc connecting the other two projected WPs (Fig. S4c). For the B-type surface, as shown in Fig. S4d-f, its iso-energy contours consist of two open Fermi arcs and keep unchanged with the variation of energies. Figs. S4g-i show the antichiral surface state dispersions at three different k_z slices for A-type (blue solid line) and B-type (red solid line) surfaces. For the A-type surface, as shown in Fig. S4g (Fig. S4i), the bulk bandgap opens and the surface states connect the projections of the lower (upper) bulk bands at $k_z = 0\pi$ ($k_z = \pi$). By contrast, the situation reverses for the B-type surface. At $k_z = 0.5\pi$, as shown in Fig. S4h, the bulk bandgap closes and the Weyl surface states connect the projections of two energy-shifted WPs for both the A-type and B-type surfaces. The numerical results calculated by the 3D modified Haldane model match well with our simulation and experimental results shown in Fig. 3 and Fig. 4 in the main text.

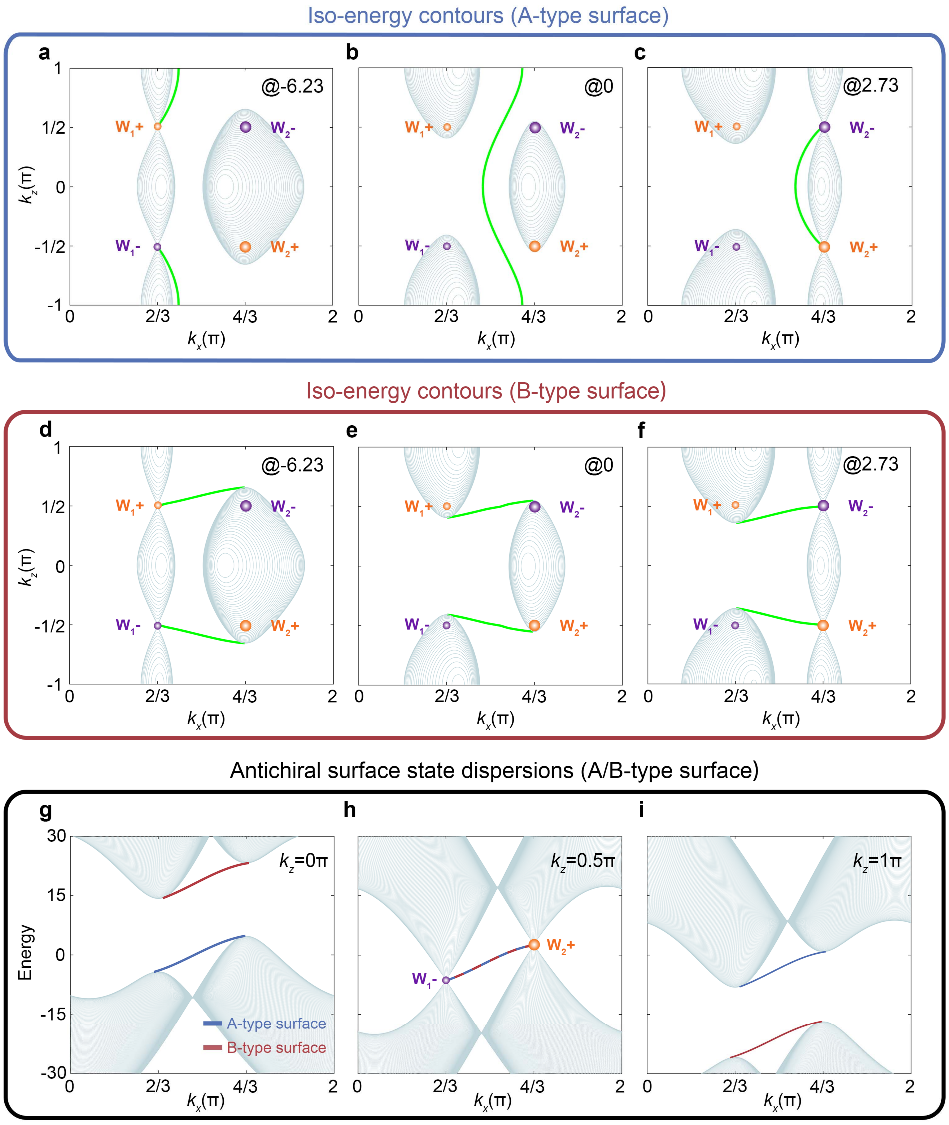

Fig. S4 | Iso-energy contours and tilted surface dispersions of antichiral surface states in 3D modified Haldane model. Calculated iso-energy contours of the topological surface states on the **a-c** A-type surface and **d-e** B-type surface at **a**, **d** E = -6.23, **b**, **e** E = 0 and **c**, **f** E = 2.73, respectively. The cyan (green) lines represent the calculated bulk (surface) dispersions, respectively. The orange and purple spheres represent the projections of the energy-shifted WPs with opposite topological charges. **g-i** Calculated surface dispersions for fixed values of **g** $k_z = 0\pi$, **h** $k_z = 0.5\pi$ and **i** $k_z = 1\pi$, respectively. The blue (red) lines indicate the A-type (B-type) surface state dispersions, respectively, and cyan regions represent the projected bulk states.

Details of the modeling of 3D magnetic Weyl photonic crystals in COMSOL simulations. We now discuss the simulation details of the bulk band structures using commercial software COMSOL Multiphysics. We first choose a hexagonal unit cell (Fig. S5a) which consists of gyromagnetic rods (red and blue colors) and permanent magnets (gray color) stacked on both sides of the perforated copper plates (yellow color). For simplicity, we replace the complementary region of copper plates and permanent magnets with air (gray color in Fig. S5b) and treat copper plates and permanent magnets as perfect electric conductor (PEC) boundary conditions (see Fig. S5c). Finally, we apply periodic boundary conditions (Fig. S5d) to the outmost boundaries to calculate the bulk band structure of the 3D magnetic Weyl photonic crystals. In the calculation of surface state dispersions, we construct a 1×20 supercell and apply periodic boundary conditions along the x and z directions, and perfect electric conductor boundaries in the y direction.

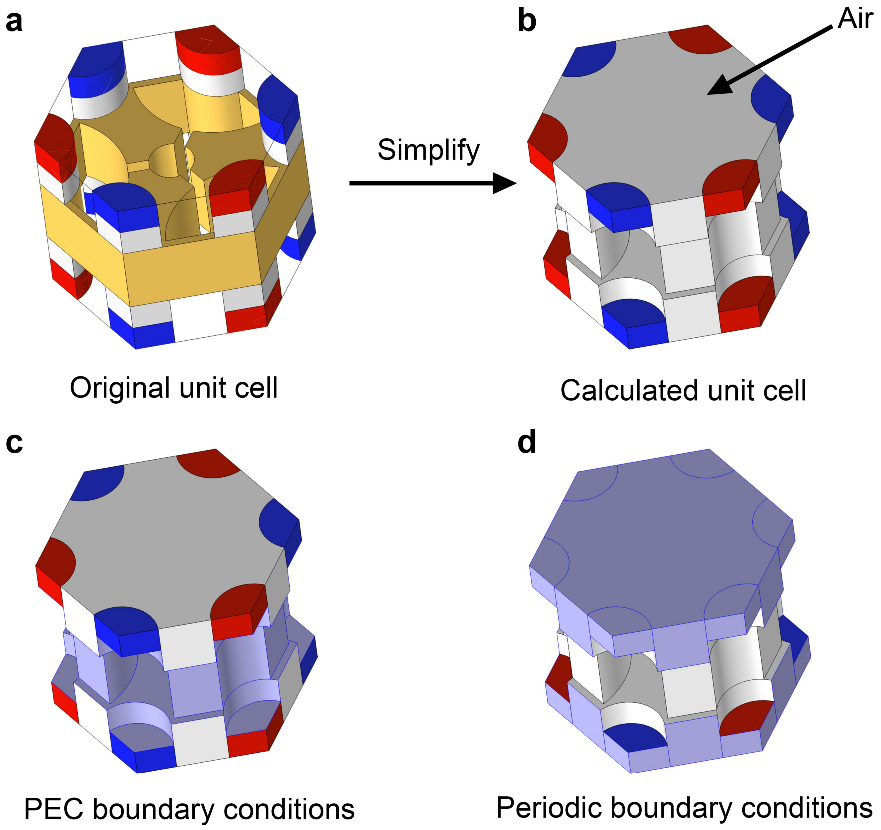

Fig. S5 | Details of modeling the 3D magnetic Weyl photonic crystal in COMSOL simulations. a Original unit cell with the real structure. **b** The simplified unit cell for calculation in COMSOL simulations. **c** Perfect electric conductor boundary conditions and **d** periodic boundary conditions for the calculated unit cell.

Topological phase transition and Berry curvature of three-dimensional (3D) modified Haldane model. For each fixed k_z, the Berry curvature can be defined in the first two-dimensional (2D) Brillouin zone (BZ) at the k_x - k_y plane. In the case of a 2D honeycomb lattice, we can choose the rhombus as the first BZ, as shown in Fig. S6a, which is defined by the reciprocal lattice vectors and discretizes the unit cell in k_i and k_j directions [*Adv. Quantum Technol.* **3**, 1900117 (2020)]. The Berry curvature can be calculated for each plaquette of the discretization using the four-point formula: for isolated bands, the integration of Berry curvature around a plaquette is given by $\phi(k)=\iint\Omega(k)ds=-\mathrm{Im}\ \log[\langle\mu_{k_1}(\mathbf{r})|\mu_{k_2}(\mathbf{r})\rangle\langle\mu_{k_2}(\mathbf{r})|\mu_{k_3}(\mathbf{r})\rangle\langle\mu_{k_3}(\mathbf{r})|\mu_{k_4}(\mathbf{r})\rangle\langle\mu_{k_4}(\mathbf{r})|\mu_{k_1}(\mathbf{r})\rangle]$, where $\mu_{k_i}(\mathbf{r})$ are the periodic functions at each corner of each plaquette. To demonstrate the topological phase transition, we first compute the Berry curvature on both sides of the WPs (k_z = ±0.5π), i.e., at k_z = ±0.45π [see Fig. S6b] and k_z = ±0.55π [see Fig. S6c], respectively. As shown in Fig. S6b, the integration of Berry curvature in the first BZ is zero, but the integration of Berry curvature in the half BZ, the valley-dependent topological index, is non-zero, indicating that the band is topologically non-trivial. The valley Chern number for k_z = ±0.45π is $C_v = C_K - C_{K'}$ = (0.47) - (-0.47) = +0.94. By contrast, the situation reverses for k_z = ±0.55π. As shown in Fig. S6c, the valley Chern number is $C_v = C_K - C_{K'}$ = (-0.47) - (0.47) = -0.94. Although the valley Chern number is not a well-defined integer, i.e., $|C_v| < 1$, the difference in the sign of the valley-dependent topological index still ensures that they are topological valley phases [*Nat. Commun.* **10**, 872 (2019)]. Fig. S6d plots the valley Chern number with the varying of k_z. It can be seen that when k_z crosses the projections of WPs (k_z = ±0.5π), the valley Chern number will change from +1 (-1) to -1 (+1). In general, the band structure with a positive valley Chern number exhibits different topological properties from that with a negative valley Chern number, indicating that topological phase transition occurs with the varying of k_z.

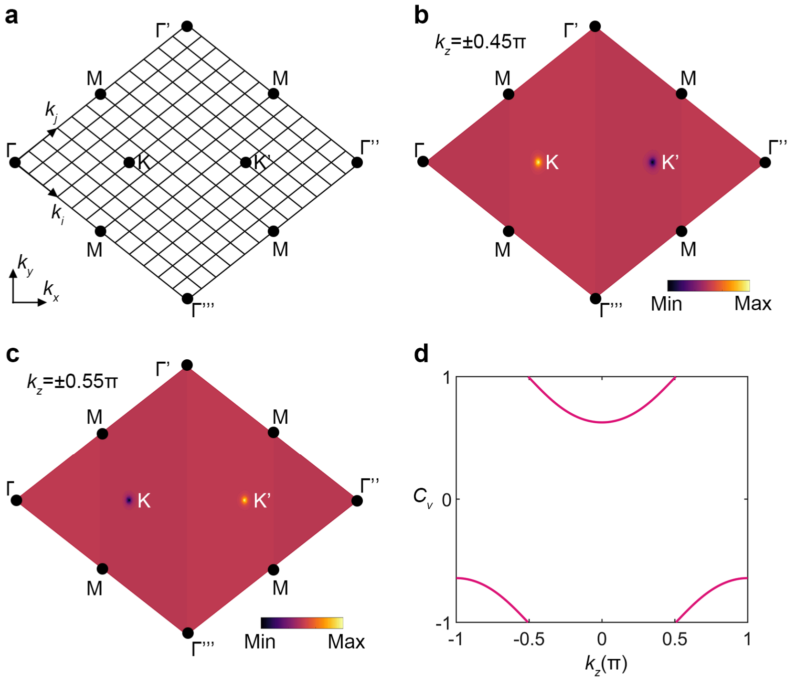

Fig. S6 | Topological phase transition and Berry curvature of 3D modified Haldane model. a Discretization of the first BZ of the honeycomb lattice. **b, c** Berry curvature distributions of 3D modified Haldane model in k_x - k_y plane with **b** $k_z = \pm 0.45\pi$ and **c** $k_z = \pm 0.55\pi$. **d** The valley Chern number with the varying of k_z.

Detailed experimental setup of magnetic Weyl photonic crystal. Fig. S7a shows a top-side view of our experimental sample which consists of 30 layers of perforated copper plates and dielectric foams inserted with about 16470 gyromagnetic rods and 32940 permanent magnets. The whole sample was assembled step by step. First, we use a gaussmeter (SHHT, HT20) to measure the polarity of magnets and use red color to mark the N-pole side and blue color to mark the S-pole side one by one. Second, we sandwich each gyromagnetic rod between two permanent magnets with the same biasing direction to form an overall uniform magnetic field and magnetize the gyromagnetic rod. Third, the sandwiched gyromagnetic rods and permanent magnets are inserted into dielectric foams according to the designed magnetic fluxes distribution on a 3D honeycomb lattice (by flipping the biasing direction of magnets to apply opposite magnetic fluxes on different sublattice sites A and B), as shown in Fig. S7b where the first copper plate on the top is shifted for visualization. Finally, we stack the dielectric foams and metallic plates layer-by-layer to assemble the whole experimental sample, as shown in Fig. S7a and Fig. 2a in the main text. To measure the surface state dispersions of the A-type and B-type surfaces, we cover the A-type and B-type surfaces (parallel to the *xz* plane) of the gyromagnetic photonic crystal with two copper claddings, acting as trivial photonic bandgap materials to localize the topological surface states, as shown in Fig. S7c. Figs. S7d-f shows the front (A-type surface), back (B-type surface) and side view of our sample, respectively.

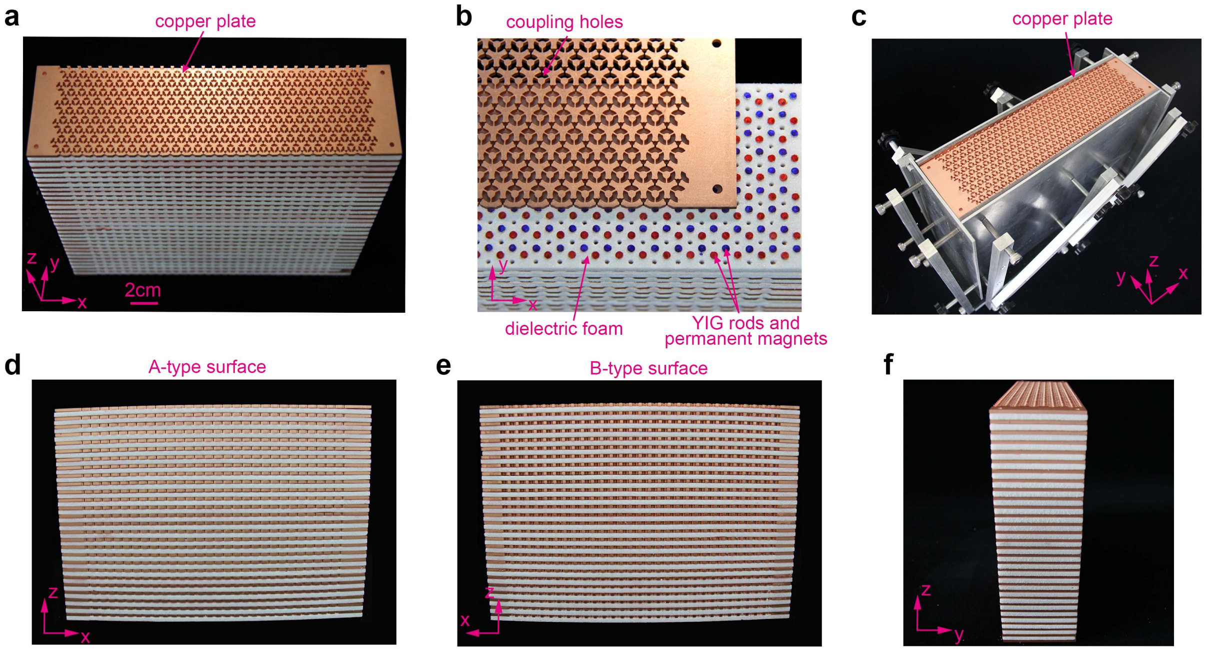

Fig. S7 | Detailed experimental setup. **a** Top side view of the experimental sample. **b** Top view of the sample with the top metallic plate shifted for visualization, where the red and blue color denote the N and S polarities of magnets. **c** Cover the A-type and B-type surfaces with two copper claddings. **d** The front view (A-type surface) of the sample. **e** The back view (B-type surface) of the sample. **f** The side view of the sample.

Surface state dispersions with two different basic boundary selections. Different from conventional topological surface states protected by Chern number, the antichiral surface states protected by the valley Chern number depend significantly on the selection of boundary since the nonzero valley Chern number only ensures the existence of surface states but does not guarantee their shapes and slopes. Consequently, the surface state dispersions will be modified by cutting the boundaries at different y planes. As shown in Fig. S8a-b, there exist two basic boundary selections, i.e., one is at the lattice point of the unit cell (Fig. S8a) and the other is at the middle of the unit cell (Fig. S8b). We have studied the former case in detail in the main text. Here, we focus on the latter case with the boundary located in the middle of the unit cell (Fig. S8b). The calculated surface state dispersions are shown in Fig. S8c-e. For the A-type surface (blue solid line), as shown in Fig. S8c and Fig. S8e, the bulk bandgap opens and the surface state connects the projections of the lower bulk bands at $k_z = 0\pi/h$, while the surface state disappears at $k_z = 1\pi/h$. The situation reverses for the B-type surface (red solid line), which disappears at $k_z = 0\pi/h$ but connects the projections of the upper bulk bands at $k_z = 1\pi/h$. At $k_z = 0.38\pi/h$, as shown in Fig. S8d, the bulk bandgap closes and the Weyl surface states (red and blue solid lines) connect the projections of two frequency-shifted WPs. For the A-type surface, the Weyl surface state (blue solid line) connects the projections of two frequency-shifted WPs through the BZ boundary ($k_x = 1\pi/a$), while for the B-type surface, the Weyl surface state (red solid line) connects the projections of two frequency-shifted WPs through the BZ center ($k_x = 0\pi/a$). Noted that all these surface states own both positive and negative group velocities at the same frequency along k_x direction, indicating that the unidirectional propagation characteristic of the surface states disappears when we cut the boundary at the middle of the unit cell.

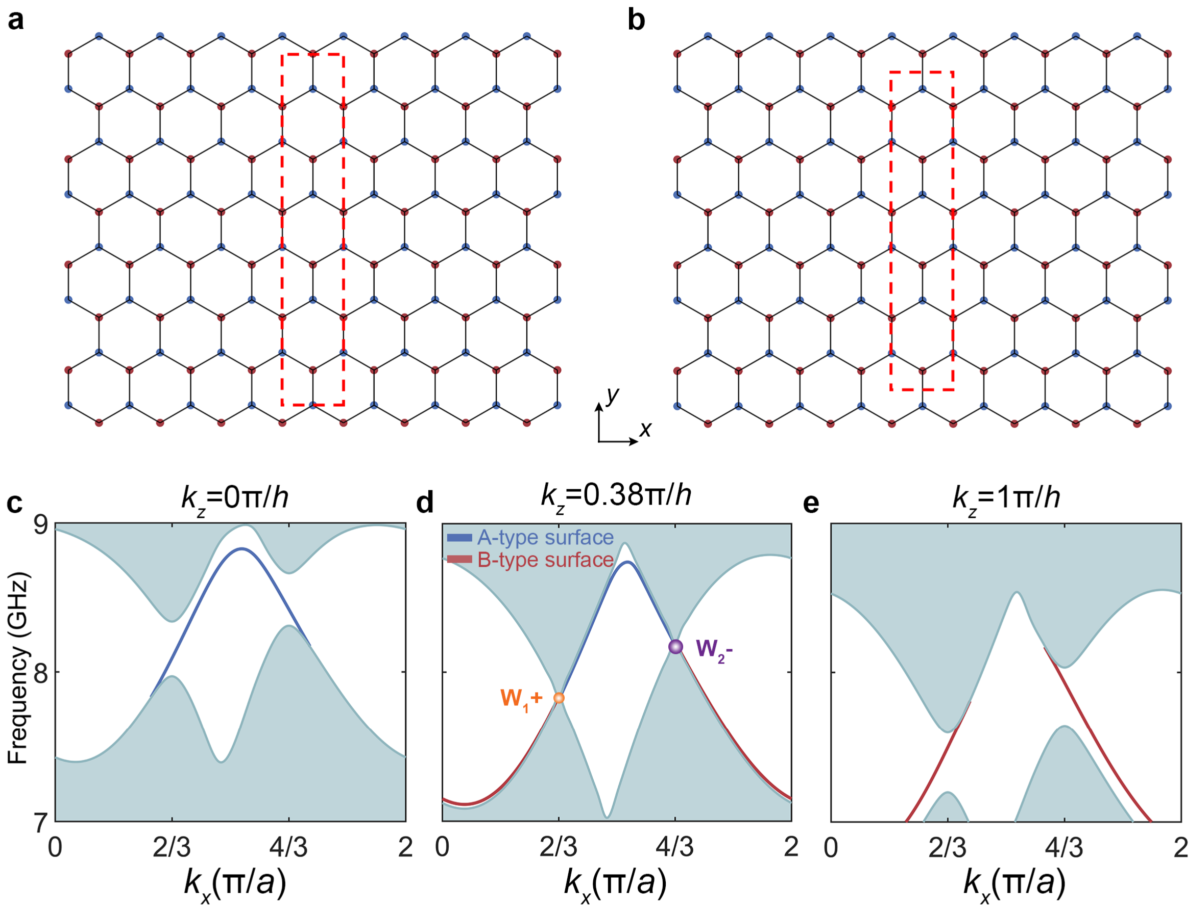

Fig. S8 | Surface state dispersions for two different basic boundary selections. a, **b** Schematics of the boundary for the ribbon cutting at **a** the lattice point of the unit cell and **b** the middle of the unit cell. Red dashed rectangles represent the top view of the supercells with different boundary selections used to calculate the surface state dispersions. **c-e** Simulated surface states dispersions for fixed values of **c** $k_z = 0\pi/h$, **d** $k_z = 0.38\pi/h$ and **e** $k_z = 1\pi/h$ with the boundary cut at the middle of the unit cell shown in **b**. Blue (red) solid lines indicate the A-type (B-type) surface state dispersion, respectively, and cyan regions represent the projected bulk states.

Unidirectional propagation of antichiral surface states. One of the most interesting properties of magnetic Weyl photonic crystals is the unidirectional propagation of topological surface states due to the time-reversal symmetry breaking. To characterize this unique property, we first perform full-wave simulations on a finite 3D magnetic Weyl photonic crystal. An electric dipole source (cyan star) is placed at the center of A-type and B-type surfaces to excite the surface states, as shown in Fig. S9a-b. For both A-type and B-type surfaces, we observe that the excited antichiral surface states always propagate rightward along the $+x$ direction, unambiguously verifying the unidirectional propagation of antichiral surface states. We then perform electromagnetic near-field imaging measurements to probe the unidirectional propagation characteristic of the antichiral surface states. We cover the frontal (A-type) and back (B-type) surfaces of the experimental sample with copper claddings, and all other surfaces with microwave absorbers. A microwave dipole antenna source (cyan star) is placed at the center of the front and back surfaces to excite the surface states. Figs. S9c-d show the measured electric field distributions of the antichiral surface states on A-type and B-type surfaces, revealing that topological antichiral surface states propagate unidirectionally along $+x$ direction for both surfaces.

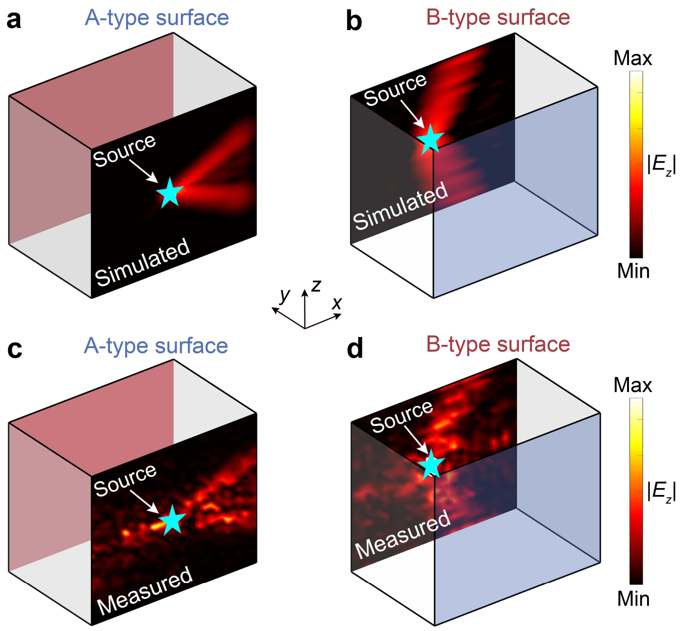

Fig. S9 | Unidirectional propagation of antichiral surface states. a, **b** Simulated and **c**, **d** measured electric field distributions of antichiral surface states at 8.02 GHz on the **a**, **c** A-type and **b**, **d** B-type surfaces, respectively, revealing the unique antichiral and unidirectional propagation properties of the antichiral surface states on opposite surfaces of the magnetic Weyl photonic crystal.

Robustness of antichiral surface states. To characterize the robustness of antichiral surface states, we insert a metallic obstacle (yellow rod) in the path of the surface states on the A-type and B-type surfaces, as shown in Fig. S10, where the surface states bypass the obstacle and continue to propagate with negligible reflection.

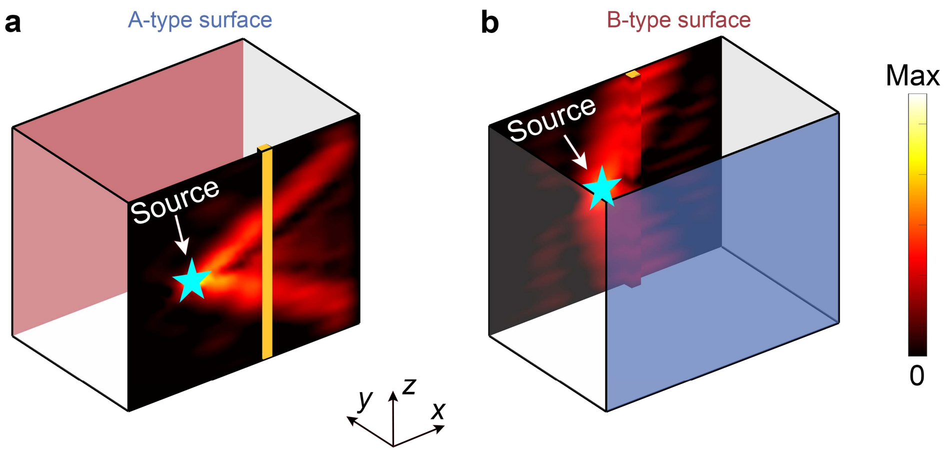

Fig. S10 | Robustness of antichiral surface states. a, **b** Simulated electric intensity distributions of the topological surface states at 8.02 GHz on **a** A-type and **b** B-type surfaces with a copper pillar (yellow rod) inserted into the photonic crystal as a metallic obstacle.

Measured electric field amplitude and phase distributions of A-type and B-type antichiral surface states. To measure the electric field distribution of the antichiral surface states on the A-type and B-type surfaces, we put a dipole source antenna at the center of the A-type and B-type surfaces to excite the surface states. Then we insert another dipole probe antenna that is fixed to a robotic arm into the air holes one by one to map the complex electric field distributions (E_z component) in the *xz* plane near the A-type and B-type surfaces. For the A-type and B-type surfaces, we plot the measured electric field amplitude ($|E_z|$) and phase ($\arg(E_z)$) of the antichiral surface states at three different frequencies (two Weyl frequencies at 7.85 GHz and 8.22 GHz and an intermediate frequency at 8.02 GHz) in Figs. S11a-f and 11g-l, respectively. After applying Fourier transform to the measured complex field distributions from real space to reciprocal space, we obtain the measured isofrequency contours in Fig. 3 and surface dispersions in Fig. 4 in the projected surface BZ in the main manuscript.

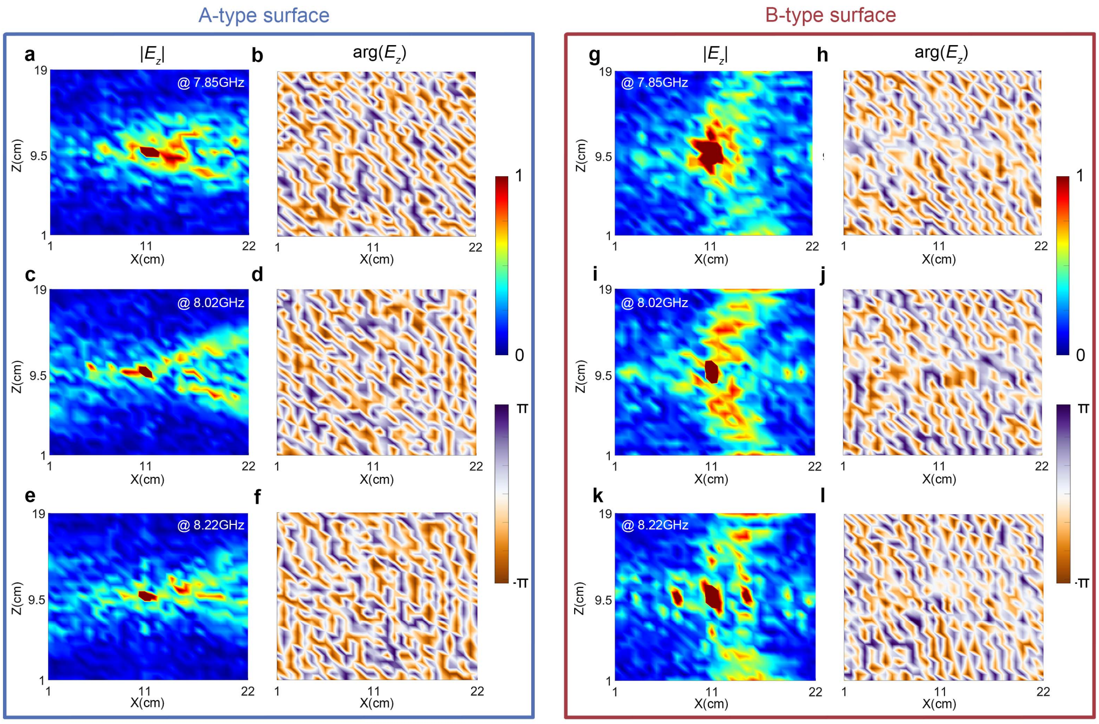

Fig. S11 | Measured *E*-field distributions of the antichiral surface states. a-f Measured amplitude and phase distributions of E_z when the surface states are excited by a point source (microwave dipole antenna) at the center of the A-type surface at 7.85 GHz, 8.02 GHz and 8.22 GHz, respectively. **g-l** Measured amplitude and phase distributions of E_z when the surface states are excited by a point source (microwave dipole antenna) at the center of the B-type surface at 7.85 GHz, 8.02 GHz and 8.22 GHz, respectively.

Detailed analysis of the degeneracy of antichiral surface states at fixed k_z. The reason for the degeneracy of two antichiral surface states at fixed $k_z = 0.38\pi/h$ is because of the mirror symmetry in the 2D modified Haldane model. In both numerical simulations and tight-binding models we can observe the degeneracy. Here we adopt tight-binding models to explain the reasons. For convenience, we rewrite the 3D modified Haldane model as

$$H_{3D} = d_x\sigma_x + d_y\sigma_y + d_z\sigma_z + d_o I, \quad (1)$$

where

$$d_x = t_1 \sum_{n=1,2,3} \cos(\mathbf{k}\cdot a_n), \quad (2)$$

$$d_y = t_1 \sum_{n=1,2,3} \sin(\mathbf{k}\cdot a_n), \quad (3)$$

$$d_z = M + (t_a - t_b)\cos(k_z h), \quad (4)$$

$$d_0 = 2t_2 \sum_{i=1,2,3} \cos(\phi - \mathbf{k}\cdot \mathrm{b}_i) + (t_a + t_b)\cos(k_z h). \quad (5)$$

σ are the Pauli matrices and I is the identity matrix. The eigenvalues of the Hamiltonian (1) are $E_\pm = d_0 \pm \sqrt{d_x^2 + d_y^2 + d_z^2}$. When we fix k_z at the projections of WPs with $d_z = 0$, this model is simplified to a 2D modified Haldane model

$$H_{2D} = d_x\sigma_x + d_y\sigma_y + d_o I. \quad (6)$$

Note that $(t_a + t_b)\cos(k_z h)$ term in (5) only shifts the energy of the band structure. In the 2D modified Haldane model, two antichiral edge states on opposite stripe edges are always degenerate because the d_0 term is proportional to the identity matrix. Compared to the pristine graphene model which has two degenerate zero edge modes on opposite stripe edges, the d_0 term does not change the degeneration of the wave functions but changes their energies which makes the edge dispersions tilt [*Phys. Rev. Lett.* 120, 086603 (2018)]. The degeneration can also be further verified by the mirror symmetry of 2D modified Haldane model in a finite lattice ($M_x H M_x^\dagger = H$). Suppose that $|\psi_1\rangle = \psi_0 e^{-\xi|y+y_0|}$ is an eigenvector of the edge state with eigenvalue E; it also exists the other eigenvector $|\psi_2\rangle = M_x|\psi_1\rangle = \psi_0 e^{-\xi|y-y_0|}$ with the same eigenvalue E, since $H(M_x|\psi\rangle) = M_x H|\psi\rangle = M_x(E|\psi\rangle) = E(M_x|\psi\rangle)$, as shown in Fig. S12. Thus $|\psi_1\rangle$ and $|\psi_2\rangle$ must be two different but degenerate antichiral edge states.

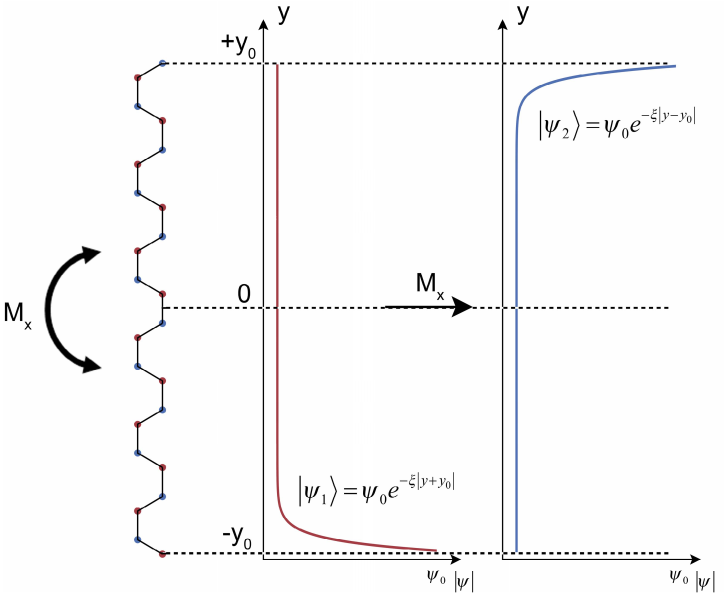

Fig. S12 | Schematic view of the 2D modified Haldane model, where $|\psi_1\rangle$ and $|\psi_2\rangle$ are two different but degenerate antichiral edge states due to the mirror symmetry.
